# The prevalence and determinants of sexual violence against young married women by husbands in rural Nepal

**DOI:** 10.1186/1756-0500-5-291

**Published:** 2012-06-13

**Authors:** Mahesh Puri, Melanie Frost, Jyotsna Tamang, Prabhat Lamichhane, Iqbal Shah

**Affiliations:** 1 Center for Research on Environment Health and Population Activities, Kusunti, P.O. Box 9626, Kathmandu, Nepal; 2 Department of International Development, University of Oxford, Oxford, United Kingdom; 3 Department of Reproductive Health and Research, World Health Organization, Geneva, Switzerland

## Abstract

**Background:**

Sexual violence within marriage is a public health and human rights issue; yet it remains a much neglected research area, especially in Nepal. This paper represents one of the first attempts to quantify the extent of sexual violence and its determinants among young married women in Nepal.

**Methods:**

A cross-sectional survey was conducted among 1,296 married women aged 15–24 years in four major ethnic groups in rural Nepal. The survey data were used to estimate the prevalence and identify determinants of sexual violence. The relative importance of different correlates of sexual violence in the past 12 months at the individual, household and community levels were examined by using a multi-level multivariate statistical approach.

**Results:**

Of the young women surveyed 46% had experienced sexual violence at some point and 31% had experienced sexual violence in the past 12 months. Women’s autonomy was found to be particularly protective against sexual violence both at the individual and community level. Women’s educational level was not found to be protective, while the educational level of the husband was found to be highly protective.

**Conclusions:**

The high prevalence of sexual violence against young women by husbands found in this study is a matter for serious concern and underscores the need for a comprehensive response by policymakers.

## Background

Gender-based violence, including intimate partner violence - physical violence perpetrated by men against their female partners - and sexual violence (SV) are worldwide public health problems associated with a wide range of negative physical, psychological, social and economic consequences for abused women themselves and their children [1-5]. Despite this myriad of adverse outcomes, few studies on intimate partner violence have been conducted in developing countries, particularly in South Asia. The experiences of young married women in particular remain largely unexplored. Though limited, research in developing countries suggests that between 2% and 48% of young women have experienced coerced sex within a formal marriage [6-14]. The World Health Organization’s multi-country study on domestic violence also found that between 4% and 57% of ever-partnered 15–19 year old women had experienced sexual violence by an intimate partner at some point [7]. Studies conducted elsewhere in the world show that sexual violence occurs virtually across all regions and cultures [15].

The factors influencing a woman’s risk of sexual violence within marriage (SVWM) are complex and not easily amenable to a simple and comprehensive conceptual framework; furthermore, no such framework currently exists. In order to guide the analysis of data, this paper builds upon the evidence reviewed in the literature. We consider that a number of interrelated factors at the individual, household and community levels act together to mutually reinforce or offset their influence on the risk of SVWM. Women’s autonomy is viewed as the most proximate determinant of SVWM, which is in turn influenced by a host of individual, couple and household factors. There are also contextual community factors, which exert their influence by changing the context within which SVWM may or may not take place. The variables included in this study cover all these levels and are explained in detail within the methods section.

Information from developing countries on the factors underlying SVWM is sparse. The limited evidence available from India and Bangladesh identified several risk factors among young women. An early and arranged marriage, lack of information on sexual matters, inability to exercise sexual and reproductive rights, unequal gender norms and lack of alternative support systems were all found to increase the vulnerability of young married women to coercive sex within marriage [16-22]. Limited evidence available from Bangladesh, India and Nepal suggests that women who marry after age 20 years are somewhat less likely to report coercive sexual experiences than women who marry earlier [20,23]. Studies of early marital sexual relations suggest that a lack of awareness of sexual matters contributes to reported traumatic experiences associated with first sex [20,23].

Few studies from developing countries have reported that alcohol consumption by men plays a significant precipitating role in domestic violence, but there is barely any evidence on the effect of alcohol consumption on sexual violence [24-26]. A study from Uganda showed that women whose partners consumed alcohol faced almost a four-fold increased risk of both physical and sexual violence relative to women whose partners did not consume alcohol [13]. Another study from South India found that expenditure on alcohol was a significant risk factor for domestic violence [27].

Empirical evidence on the broader contextual and community-level factors shaping risk of SVWM is even sparser. A study conducted in Bangladesh showed that the effect of women’s status on violence was context specific. In more culturally conservative areas, higher individual women’s autonomy and short- term membership in savings and credit groups were both associated with significantly elevated risks of violence while community-level factors were unrelated to violence. In less culturally conservative areas, meanwhile, individual-level women’s status indicators were unrelated to the risk of violence, and community-level measures of women’s status were associated with a significantly lower risk of violence [28]. Another study conducted in four states of India showed no statistically significant relationship between women’s status and sexual violence among young women. Whether or not improvements in women’s status and autonomy contribute to reductions in sexual violence thus remains an unresolved issue [11].

Patriarchal family structure, cultural, social and religious patterns in Nepal all enforce the lower status of women in family and society; these may act as a catalyst for sexual violence [29]. Moreover, Nepali women are generally shy and introverted on matters relating to sex and husbands generally see no problem in exercising some force when they desire sexual intercourse [30]. Sex education in school and counselling services related to sex and sexuality are still taboo subjects [31]. Moreover, many women in Nepal hold the view that it is in their *dharma* (defined as religion, moral duty and universal law) to be obedient, respectful and pleasing to their husbands [32,33].

In 2009, the Government of Nepal passed a comprehensive law on gender-based violence which made it a criminal offence for a husband to have forced sex with his wife. The new law stipulated that the punishment for such an offence range from a fine to six months in prison depending on the type of sexual violence [34]. In practice, however, this law is rarely enforced strictly. Moreover, the majority of Nepalese people, including local authorities, local police and other agencies that supposedly deal with gender-based violence, are still unaware of its existence [35].

In addition, there is very limited evidence on the prevalence of violence against women or its dynamics in Nepal and sexual violence in particular has been neglected. A woman is defined as having experienced SVWM if she has suffered one or more of the following: (a) forced sexual intercourse; (b) being forced to do something sexual that was found by her to be degrading or humiliating; (c) fearing the consequences of refusing sex; and/or (d) being threatened by her husband that he would leave or go to another woman if she refused him sex. A study conducted among married women of reproductive age in four districts of Nepal found that nearly 58% of women had experienced some form of sexual coercion by their husbands. The study also showed that literate women were less likely to experience sexual coercion, as were women who had some degree of control over decisions regarding their own healthcare [36]. Likewise, another study undertaken to examine experience of violence among young married women in rural Nepal revealed that nearly half (46.2%) reported ever experiencing sexual violence and 31.3% reported having experienced sexual violence in the past 12 months. Results in the study indicated that women’s autonomy and inter-spousal communication were significant predictors of violence [36]. However, the study had combined both physical and sexual violence as a measure of violence and, importantly, neither paper [36,37] took account of community level factors.

To better understand, the role of individual and community level factors in women’s experience of SVWM, this paper uses cross-sectional survey data which has already been analysed to examine women’s status and ‘violence in general’ [37]. More specifically, we examine whether women’s autonomy, level of education, inter-spousal communication, family support, traditional cultural practices (early and arranged marriage) and selected men’s characteristics (alcohol use, multiple partners, education, occupation etc.) are associated with SVWM. In addition, the paper examines the role of community level indicators.

This paper aims to contribute to the limited body of population-based evidence on the prevalence and determinants of SVWM among young married women in developing countries and also to inform policies and programs for its prevention in Nepal.

## Methods

### Data

Data for this paper come from a cross-sectional survey, carried out in four districts (Dolkha, Sindhupalchowk, Dang and Kapilvastu) among four major ethnic groups (Brahmin/Chhetri, Tamang, Tharu and Muslims) by the Centre for Research on Environment Health and Population Activities (CREHPA) in 2009. These districts were selected because they have a high concentration of the study’s ethnic communities (more than 50%of the total population in the districts) and also represent geographic variation (two hill and two terai or plains).

The survey interviewed 1,296 married women aged 15 to 24 years old, selected by using a two-stage systematic random sampling technique. In the first stage, 48 clusters in the selected districts were chosen using the population proportionate to size sampling technique. In the second stage, sample households were selected from the sampled clusters. Before selection, a list of households was updated with the help of community leaders and this list served as a sampling frame for selecting households. A systematic random sampling technique was applied to the list to select 27 households from each cluster. After selecting a house, a short screening questionnaire was administered to the head of the household in order to identify eligible respondents. A total of 5,080 households were visited to identify eligible respondents. Although all the eligible respondents (a total of 1,811 married women aged 15–24 years) were identified in the households visited, the desired sample size for the study (i.e. 1,296) was achieved using systematic random sampling and the respondents were interviewed. Only respondents who gave their informed consent to participate in the study were included. In the case of non-availability of eligible respondents, interviews were terminated after completing the short screening household questionnaire. In households with more than one eligible respondent, one respondent was selected randomly for interview. Interviews were conducted individually at a convenient location for the respondents, usually away from their homes, by well-trained female Nepali interviewers. During the field study, two authors of this article visited the study sites and supervised the interviewers to ensure interview quality and the respondent’s privacy.

A structured questionnaire was used for the face-to face individual interviews. Both the format and the questions were based on the WHO Multi-country Study on Women’s Health and Domestic Violence against Women, but were adapted to the local setting and study population. The questionnaire was first developed in English and then translated into Nepali. It was pre-tested among similar population subgroups outside the selected study areas and necessary modifications were made.

The core protocol and research instruments were approved by the Nepal Health Research Council (the Nepal government’s ethical approval agency) and the World Health Organization’s (WHO) Research Ethics Review Committees (ERC). Participants’ verbal consent was obtained regarding their participation in the study. Confidentiality of information was ensured by removing personal identification from the data and by securing access to all data and information. Interviewers orally provided the name and address of organizations that deal with sexual violence and conflicts within marriage to all women. Moreover, if any respondent reported having experienced violence and wished to seek help (requiring counselling or other services), the interviewers facilitated their access to an appropriate service facility or referred them to their nearest health centre. None of the respondents eligible for the study refused to be interviewed.

### Dependent variable

The dependent variable in the multivariate analysis was whether or not the individual woman reported having experienced sexual violence by her husband in the 12 months preceding the interview. This variable was based on a series of questions that were asked in the survey – these questions were:

Did your husband ever physically force you to have sexual intercourse with him even when you did not want to?

Was there ever a time when you were afraid to say ‘no’ to sex with your husband?

Did your husband ever threaten you that if you didn’t have sex with him he would leave or go to another woman?

Did your husband ever force you to do something sexual that you found degrading or humiliating?

Women who answered affirmatively to any of these questions were counted as having experienced SVWM. Women who answered yes to any of these questions were also asked if those experiences had occurred at all in the past 12 months, and if so how frequently. Women who responded affirmatively that they had experienced one or more of these acts in the past 12 months were categorized as having experienced sexual violence in the 12 months preceding the interview.

### Independent variables

At the individual level, apart from typical background characteristics (age, number of living children, occupation, education of both the woman and her husband), variables reflecting women’s autonomy, involvement in community groups and access to mass media were considered. Also, variables reflecting young women’s transition to marriage and sexual life as indicated by age at marriage, type of marriage and knowledge about sex before marriage were included in the analysis. At the couple level, we included a variable reflecting inter-spousal communications, while at the household level an asset index was included. At the community-level, women’s autonomy (discussed further below), women’s education and men’s education were included.

Independent variables that were thought to be conceptually important (based on the literature review and the conceptual framework) were initially included in the analysis. Variables in the multivariate analysis were included on the basis of forward model selection. The coding categories of most of the variables included in the analysis are self-explanatory. Nevertheless, details on the key individual level variables have been presented in Table 1.

**Table 1:** Description of measures

**Variable**	**Coding**	**Description**
Household and individual level		
Age of women at time of survey	Continuous	Completed age of woman at the time of survey in years
Age of woman at marriage	1 = Under 15 years	Age of woman at marriage in years
	2 = 15–17 years	
	3 = 18 and over	
Type of marriage	1 = Love marriage	Self-reported. A love marriage is a marriage in which participant and her husband decided themselves to get married.
	2 = Arranged marriage	An arranged marriage is one in which family members decided that the couple would get married.
Sex composition of the child	0 = No child	Sex composition of the living child
	1 = At least one son	
	2 = Only daughter	
Caste/ethnicity	1 = Brahmin/Chhetri	Caste/Ethnicity of the woman
	2 = Tamang	
	3 = Tharu	
	4 = Muslims	
Woman’s education	0 = Illiterate/never been to school	Number of years of education completed by the women
	1 = Literate up to 8 class/NFE	
	2 = Secondary	
	3 = Higher secondary and above	
Women’s occupation	1 = Do not earn/housewife	Occupation of the woman at the time of survey
	2 = Agriculture/daily wage/livestock	
	3 = Other employment/small business	
Membership in community groups	0 = No	Membership of a community group
	1 = Yes	
Current age of husband	1 = 15–24 years	Completed age of husband at the time of survey
	2 = 25–34 years	
	3 = 35 and above years	
Husband’s level of education	0 = Illiterate/never been to school	Number of years of education completed by the women’s husband
	1 = Literate up to 8 class/NFE, 2 = Secondary	
	3 = Higher secondary and above	
Husband’s occupation	1 = Unemployed	Occupation of the woman’s husband at the time of survey
	2 = Agriculture	
	3 = Daily wage labor	
	4 = Service	
	5 = Foreign employment	
	6 = Business	
Husband’s alcohol consumption	1 = Often	Frequency of alcohol consumption of husband
	2 = Sometimes	
	3 = Never/rarely	

Some of the variables were generated from the individual data. The women’s autonomy variable was constructed through latent class analysis [38,39]. We considered women’s autonomy as a latent construct because it is not directly observable and it is most appropriately measured through multiple observable manifest variables. The advantage of latent class analysis is that it probabilistically categorizes women into latent classes on the basis of the distribution of their responses to the specified manifest variables. In order to construct an index of women’s autonomy, we used 14 questions (Table 2) reflecting three key dimensions of women’s autonomy drawing on work by Jejeebhoy (2000) and Koenig (2003) – these three dimensions were autonomy/mobility, familial decision making power and control of resources [13,40]. The possible answers for questions relating to decision-making were ‘respondent’, ‘husband’, ‘respondent and her husband’, mother-in-laws’, father-in-laws’ and other. The first two responses were condensed into “self or joint” and the last three to “others”. The question on easiness in sharing feeling with husband was dichotomized into “very easy/easy” and “difficult/very difficult”. Rest of the questions were coded as “Yes” or “No”.

**Table 2:** Description of indices

**Index**	**Coding**	**Questions used in survey**
Women’s autonomy	1 = High autonomy	· Generally who makes decisions about purchasing domestic goods?
	2 = Medium autonomy	
	3 = Low autonomy	· Generally who makes decisions about you giving birth or not?
		· Generally who makes decisions about the number of children you should have?
		· Generally who makes decisions about your using or not using a family planning method?
		· Are you able to visit friends or relatives without permission of your husband or other family members
		· Are you able to visit the health centre or hospital without permission of your husband or other family members?
		· Are you able to visit any organization without permission of your husband or other family members?
		· Are you able to hold any group membership in the community without permission of your husband or other family members?
		· Are you able to spend money without permission of your husband or other family members?
		· Do you earn something to contribute to the family income?
		· In general do you talk to your husband about money matters?
		· In general do you talk to your husband about things that worry you?
		· In general do you talk to your husband about things that worry him?
		How easy do you feel in sharing your feelings with your husband?
Inter-Spousal Communication	0 = None	In general, would you say you talk to your (current or most recent) husband/partner about the the following topics:
	1 = Discussed on 1–3 matters	· Money matters
	2 = Discussed on 4 or more matters	· Things that have happened to him in the day
		· Things that happen to you during the day
		· Things that worry you
		· Your feelings, love
		· Things that worry him
		His feelings, love
		Whether or not the household have
Household wealth index	1 = Poorest	· electricity
	2 = Poor	· a radio
	3 = Middle	· a television
	4 = Rich	· a telephone
	5 = Richest	· a gas stove
		· a kerosene stove
		· tap water,
		· a bicycle or a motorbike or tractor
		· type of floor of house
		· type of roof of house
		number of rooms
Exposure to media	0 = No/rare exposure	· How often do you read newspapers and magazines?
	1 = Frequent exposure to all three media	· How often do you listen to the radio?
		· How often do you view television?

**Table 3:** Distribution of young married women aged 15–24 years by selected background characteristics: Rural Nepal, 2009

**Background characteristics**	**Number**	**%**
Age of women	79	6.1
15–17 years	398	30.7
18–20 years	819	63.2
21–24 years		
Median age at marriage(years)	17.0	
Duration of marriage		
Less than one year	127	9.8
1–2 years	318	24.5
3–4 years	320	24.7
5 years and above	521	41.0
Mean duration of marriage	4.0	
Type of marriage		
Arranged	908	70.1
Love marriage	388	29.9
Number of living children		
0	352	27.2
1	502	38.7
2	326	*25.2*
3 or more	116	8.9
Caste/Ethnicity		
Brahmin/Chhetri	324	25.0
Tamang	324	25.0
Tharu	324	25.0
Muslim	324	25.0
Woman’s level of education		
Illiterate	371	28.6
Literate up primary	664	51.2
Secondary	212	16.4
Higher secondary and above	49	3.8
Woman’s occupation		
Do not earn/housewife	1019	78.6
Agriculture/poultry/daily wages	220	17.0
Other employment (Service/small business)	57	4.4
Women’s autonomy		
High	488	37.7
Moderate	484	37.3
Low	324	25.0
Household wealth index		
Poorest	250	19.3
Poor	265	24.5
Middle	259	20.0
Rich	261	20.1
Richest	261	20.1
Membership in community groups		
Yes	480	37.0
No	816	63.0
Media exposure		
No/rare	742	57.3
Frequent	554	42.7
Husband’s education		
Illiterate	180	13.9
Literate up to primary	695	53.6
Secondary	282	21.8
Higher secondary and above	139	10.7
Total	1296	100.0

**Table 4:** Percentage of young married women reporting experiences of sexual violence by their selected individual characteristics: Rural Nepal, 2009

**Background characteristics**	**% reporting life time experience of SV**	**% reporting experience of SV in last 12 months**	**N**
Current age of women		***	
15–17 years	51.9	45.6	79
18–20 years	47.5	35.9	398
21–24 years	45.1	27.6	819
Age at marriage	**		
Les than 15	59.3	34.1	91
15–17	48.1	32.1	672
18 and over	41.6	29.6	533
Duration of marriage		***	
Less than 1 year	52.0	48.8	127
1–2 years	42.5	32.7	318
2–4 years	45.0	28.4	320
5 and more years	47.8	27.9	531
Type of marriage			
Arranged	47.8	31.4	908
Love marriage	42.5	30.9	388
Sex composition of living child		***	
Childless	46.9	40.6	352
At least one son	46.1	28.0	601
Only daughter	42.3	27.4	343
Woman’s level of education		**	
Illiterate	46.4	34.8	371
Literate up primary (up to 8 years)	47.9	32.5	664
Secondary (9–10 years)	42.9	24.1	212
Higher secondary and above (11 years and above)	36.7	18.4	49
Woman’s occupation		**	
Do not earn/housewife	46.3	30.2	1019
Agriculture/poultry/daily wages	47.7	39.6	220
Other employment (Service/small business)	38.6	17.5	57
Knowledge of sex prior to marriage			
Yes	46.9	33.5	698
No	45.5	28.6	598
Media exposure		*	
No/rare	47.3	33.6	742
Frequent	44.8	28.2	554
Women’ autonomy	***	***	
High	38.9	23.4	488
Moderate	49.2	32.0	484
Low	52.8	42.0	324
Total	46.2	31.3	1296

Chi-square test of significant at * p ≤ 0.05; ** p ≤ 0.01; ** *p ≤ 0.001.

**Table 5:** Percentage of young married women reporting experiences of sexual violence by their selected household and interpersonal characteristics: Rural Nepal, 2009

**Background characteristics**	**% reporting life time experience of SV**	**% reporting experience of SV in last 12 months**	**N**
Household asset index		**	
Poorest	46.0	25.2	250
Poor	46.4	28.3	265
Middle	46.0	28.2	259
Rich	48.7	35.3	261
Richest	44.1	39.1	261
Caste/Ethnicity		***	
Brahmin/Chhetri	48.2	19.8	324
Tamang	40.4	21.9	324
Tharu	48.2	40.4	324
Muslim	48.2	42.9	324
Frequency of contact with maternal family	**	***	
At least once a week	43.3	29.5	349
Once a month	42.5	25.8	562
Rare/never	54.3	40.8	385
Membership in community groups		***	
Yes	47.3	34.9	480
No	44.4	25.9	816
Inter-spousal communication	***	***	
None	73.2	69.6	56
Discussed on 1–3 matters	55.5	48.7	119
Discussed on 4–7 matters	43.9	27.5	1121
Total	46.2	31.3	1296

Chi-square test of significant at * p ≤ 0.05; ** p ≤ 0.01; ** *p ≤ 0.001.

We used posterior probabilities of class membership in order to assign each woman to a modal class [41]. Our analysis included three latent classes for women’s autonomy. Women belonging to class 1 were most likely to be able to make their own decisions concerning allocation of household resources, contraception, medication, visits to their maternal family and group membership as well as being most likely to be able to communicate freely with their husbands on a variety of topics. Women belonging to class 2 were generally not involved with household decision making, except in terms of contraception and tended to have some restrictions on things like becoming a member of community groups, but they still did well in terms of communication and tended to be free to visit friends and family. Women in class 3 were the least likely to be involved with any kind of family decision making, even in terms of their own health; women in this group also felt less able to communicate with their husband.

Similarly, the inter-spousal communication variable was generated by using seven questions (Table 2). Affirmative responses on these inter-spousal communications questions were summed up and categorised into “none/low” communication on any matters (discussed no matters), medium (discussed 1–3 matters) and high (discussed 4 or more matters). Likewise, exposure to mass media was based on woman’s frequency of exposure to newspapers and magazines, radios and television which was then dichotomized into “no/rare” and frequent exposure to all three media (Table 2).

The household wealth index variable was constructed using 13 indicators of household possessions. The household possession or dwelling characteristics used for creating household wealth index has been listed in Table 2. Each asset was assigned a weight (factor score) generated through Principal Component Analysis (PCA) and the resulting asset scores were standardized in relation to a normal distribution with a mean of zero and a standard deviation of one [42,43]. Each household was then assigned a score for each asset and the scores were summed for each household; women were ranked according to the score of the households in which they resided. The sample was then divided into quintiles from one (poorest) to five (richest).

The community-level variables were generated by taking the average of individual responses at the cluster level. The cluster level variables were the percentage of women with a high level of autonomy, the percent of women with any education and the percent of husbands with any education. These variables were created from the individual level data.

### Statistical analysis

Bi-variate and multivariate statistical analyses were carried out. Chi-squared tests were performed in order to explore bi-variate associations. The bi-variate associations were tested on the variables that were important for theoretical reasons and on the basis of evidence from other countries. Multivariate analysis was then conducted in order to control for possible confounding.

Due to the binary nature of the dependent variable (having experienced sexual violence in the past 12 months), logistic regression was used. A multilevel model was used to account for the clustered nature of the data. A binary logistic regression model is presented alongside a multilevel logistic regression (wherein we introduce a random intercept in the linear predictor in order to account for the clustered nature of the data and allow for the inclusion of community level variables). Both models are presented in order to explore how community level variables affect the risk of SVWM, but also to show that the results of the logistic regression are robust to the inclusion of community effects as well as clustering within the data. The multilevel model presented uses a forward modelling selection procedure and is more parsimonious than the single level model, which includes all the variables thought to be of interest for comparison purposes. The random intercept accounts for unobserved heterogeneity within clusters. The model is specified thus:

(1) $$logit\left\{Pr\left(y_{\mathrm{ij}}=1|x_{\mathrm{ij}}\varsigma_{j}\right)\right\}=\beta_{1}+\beta_{2}x_{2j}+\beta_{3}x_{3ij}+\varsigma_{j}$$

This model includes covariates at the individual and household-level and also aggregate covariates at the cluster level, which are there to account for community effects. During the process of analysis, multi-collinearity between the variables was assessed and this resulted in dropping the variable on religion as it was highly correlated with caste/ethnicity. We also dropped the variables on husband’s drug use as it was correlated with husband’s alcohol intake.

## Results

### Characteristics of respondents

One-third of respondents were less than 20 years old. The median age at marriage was 17 years and 70% had an “arranged” marriage. The mean duration of marriage was four years and about 10 percent of respondents had been married for less than one year. Twenty-seven percent of women had no living children. About 29% of women had no schooling while 51% had completed 8 years of schooling. Nearly 80% were not working for money. A quarter of women were in the category of “low autonomy”. Over half of women (57%) had no/rare exposure to the mass media. About half of the husbands were literate up to primary school level (54%) and nearly one-third had secondary or higher level education (33%).

### Prevalence of sexual violence

Overall, 46% of young married women reported that they had experienced sexual violence by their husband at some point and 31% reported having experienced sexual violence in the last 12 months.

### Determinants of sexual violence

To inform an optimal multivariate analysis and to consider the simple (bivariate) associations between background variables and SVWM, chid-squared tests for association were used.

Younger women were more likely to have experienced sexual violence in the last 12 months but there was no significant difference by age in lifetime experience. This implies that sexual violence is more common at younger ages since for younger women the last 12 months will be the majority of their lifetime experience. Similarly, recent sexual violence decreased with an increase in the duration of marriage, but lifetime experience did not, indicating that SVWM is most common at the beginning of a marriage. Women with children were less likely to experience sexual violence in the last 12 months, particularly those with a living son.

Differences by educational level were substantial with illiterate women and women with illiterate husbands being most likely to have experienced SVWM in the last 12 months. In line with this, those women who did not have a cash income or who were employed in lower status jobs (agriculture, poultry, daily wage labour etc.) were most likely to experience sexual violence compared to those women who were involved in service or small business. Women with the lowest autonomy also experienced the highest levels of violence (42% versus 23% with the highest autonomy in the last 12 months).

Women who could rely on support from their natal family were less likely to have experienced sexual violence, indicating the importance of social networks; this association was strongest in relation to sexual violence experienced in the 12 months prior to the survey. In contrast, women who were a member of any community organisation/groups were actually more likely to have experienced sexual violence, especially in the last 12 months. This is somewhat surprising, but probably the result of another confounding variable. Women who had no communication with their husband on family or personal matters were more likely to experience sexual violence.

Moreover, a variety of the husband’s characteristics affected a woman’s likelihood of being exposed to sexual violence (results not shown in table). Having a husband with a high educational level was found to be protective as was having a husband working in service, business or foreign employment. The highest prevalence was associated with a husband who was unemployed or a daily wage labourer. Husband’s age worked in the opposite direction to women’s age as older husbands (over 35) were more highly associated with incidence of sexual violence. Women with husbands who used drugs, often consumed alcohol and had multiple casual partners were unsurprisingly at greater risk of sexual violence.

### Multivariate analysis

The relationships observed in the bi-variate analysis were reassessed by using multivariate analysis to identify statistically significant determinants, adjusting for the confounding effects of other factors. In the multivariate analysis, we considered the determinants of SVWM in the 12 months prior to the survey. Two different models are presented – model 1 is a logistic regression, which includes just the household and individual-level factors, while model 2 is a multilevel model including a more parsimonious version of the logistic regression as well as community-level variables. Results are presented in Table 6.

**Table 6:** Logistic regression models of determinants of sexual violence against young women in the past 12 months: Rural Nepal, 2009

**Variables**	**Model 1**			**Model 2**		
	**Single level**			**Random effects**		
	**Odds Ratio**		**SE**	**Odds Ratio**		**SE**
*Woman and household level variables*						
Age at time of survey	0.900	***	0.028	0.902	***	0.028
Woman has no children	2.000	**	0.440	1.994	**	0.438
No knowledge of sex prior to marriage	0.712	*	0.111	0.744	+	0.115
Frequent media exposure	0.737	+	0.130	0.728	+	0.126
Woman’s autonomy (ref: low)						
Medium	0.838		0.150	0.827		0.147
High	0.459	***	0.098	0.403	***	0.088
Caste/Ethnicity (ref: Brahmin/Chhetri)						
Tamang	0.803		0.221	0.642	**	0.171
Tharu	1.439		0.418	1.216		0.332
Muslim	1.764	+	0.560	3.930	***	1.532
Never watched a pornographic film	0.507	**	0.109	0.496	**	0.106
Contact with maternal family (ref: at least weekly)						
Once a month	0.703	*	0.124	0.717		0.126
Rare/never	1.343		0.257	1.363		0.259
Age of woman at marriage (ref: under 15)						
15–17	1.086		0.306	-		-
18+	1.046		0.316	-		-
Woman’s education (ref: illiterate)						
Literate up to grade 8	1.381		0.242	-		-
Secondary	1.347		0.387	-		-
Above Secondary	1.232		0.627	-		-
Woman’s occupation (ref: none)						
Agriculture/poultry/daily wage	1.331		0.28	-		-
Other employment	0.745		0.297	-		-
Member of community group/groups	0.924		0.166	-		-
HH asset index (ref: poorest)						
Poor	1.284		0.308	-		-
Middle	1.1		0.274	-		-
Rich	1.442		0.381	-		-
Richest	1.365		0.406	-		-
*Husband level variables*						
Age of husband (ref: 15–24)						
25–34	1.308		0.214	1.341		0.219
35 and above	3.282	**	1.277	3.304	***	1.286
Husband’s education (ref: illiterate)						
Literate up to grade 8	0.712		0.149	0.703	**	0.147
Secondary	0.402	***	0.104	0.395	***	0.101
Above Secondary	0.420	**	0.142	0.435	*	0.144
Husband’s occupation (ref: unemployed)						
Agriculture	0.604		0.198	0.58		0.189
Daily wage labour	0.655		0.225	0.591		0.202
Service	0.738		0.248	0.662		0.222
Foreign employment	0.25	***	0.088	0.233	***	0.082
Business	0.663		0.261	0.631		0.248
Husband’s alcohol consumption (ref: often)						
Sometimes	0.645	*	0.140	0.609	*	0.133
Never/rarely	0.435	***	0.102	0.428	***	0.101
Husband has casual partners	2.901	***	0.678	2.822	***	0.659
Inter-Spousal communication (ref: low)						
Medium	0.446	*	0.173	0.439	*	0.169
High	0.2	***	0.069	0.201	***	0.068
*Community level variables*						
% of women with high autonomy	-		-	0.632	**	0.113
% of women with education	-		-	1.01		0.014
% of men with education	-		-	0.969		0.019

+ p ≤ 0.10; *p ≤ 0.05; **p ≤ 0.01; ***p ≤ 0.001.

As shown in the bi-variate analysis, younger women were more likely to have experienced SVWM, adjusting for the confounding effects of other variables. Women with older husbands (over 35) also had an increased risk of SVWM, indicating that a large age gap between husband and wife could cause SVWM. Muslim women were found at significantly higher risk of SVWM compared with Brahmin/Chhetri women. A highly educated husband was a protective factor against experiencing SVWM; the odds of a woman with a husband educated to higher secondary level or above experiencing sexual violence were about 60% lower than for women whose husbands were illiterate or had no formal education. Husband’s occupation did not have much of an effect, though having a husband in foreign employment was found to be protective. It should be noted that the characteristics of the woman’s husband were generally found to be the most significant factors in determining whether the woman had experienced sexual violence.

Being childless proved to be a significant risk factor with childless women having odds of experiencing sexual violence that were about twice as high as women with children. As expected, women’s status/autonomy was found to be significant and protective. Women with high autonomy had odds of experiencing sexual violence that were about 50 or 60% lower than those with low autonomy. Inter-spousal communication was also found to be significant with high levels of communication being associated with much lower levels of violence (the odds were 80% lower for women with high inter-spousal communication levels compared to those with low communication levels). Women whose husbands had casual sexual partners or more than one wife (as reported by women) were more than twice as likely as others to have experienced SVWM in the last 12 months.

Model 2 includes three community level variables – the percentage of women with high autonomy, the percentage of women with any education and the percentage of husbands with any education. The variables found to be statistically insignificant in Model 1 were excluded from model 2. Overall a similar pattern emerged. However, some of the previously discussed individual and family-level effects were modified with the addition of community-level indicators. For example, the effect of frequency of contact with maternal family was weakened.

Of the three community level variables included only one was found to be statistically significant and this was the community level of women’s autonomy. Women living in a community where most women had a high level of autonomy were much less likely to experience sexual violence, even if they had low autonomy themselves. Given that autonomy is significantly protective at both the individual and community level improving women’s autonomy might be one of the most important paths to reducing the high levels of sexual violence found in Nepal.

## Discussion

This study represents one of the first efforts of its kind to quantify the extent of SVWM among young married women in rural Nepal. Given the wide variability in sampling procedures, interview conditions and question wording, any comparison to other societies is difficult to make. However, the prevalence of SVWM among young married women in Nepal appears similar to those in other developing countries and especially South Asia [10,11].

Many risk factors are also similar. However, the analysis carried out in this paper has highlighted some new findings. It was found that women’s education and occupation were unrelated to SVWM, as was the household wealth (as measured by an asset index). Furthermore, no evidence was found that community-level education of either men or women affected the risk of SVWM. On the other hand women’s autonomy was found to be extremely important both at the individual level and the community level. Higher levels of individual women’s autonomy were significantly associated with lower risks of SVWM. This could be due to the fact that women’s greater overall mobility, decision making power and control of resources may reinforce or solidify nascent normative changes in gender relations and rules governing women’s behaviour within the family and the community more broadly. These changes bring with them attendant changes in men’s behaviour vis-à-vis women, including the *presumed* right of husbands to resort to sexual violence with their wives [28].

Husband’s education and inter-spousal communication were also statistically significant. This implies that one of the most important ways to curtail SVWM is to involve and educate men on the issues. Indeed, variables associated with the husband tended to have more of an effect than variables associated with just the woman’s characteristics. Men need to be taught about women’s rights and to respect their wives and also learn to communicate with them. Aside from educating men on both preventing sexual violence and respecting women, it is important to continue to try and raise each woman’s autonomy/status in order to reduce SVWM. The increased risk of SVWM with increased consumption of alcohol by the husband is not a new finding. This study supports the earlier findings from Nepal and other developing countries which reported that alcohol use plays a significant precipitating role in sexual violence [13,24,26-28,36]. Therefore, any campaign against alcohol use should also cover the issue of sexual violence, among other issues. Additionally, women with husbands who have multiple casual partners or wives were at especially high risk.

This study showed some surprising findings, especially in regards to education. One expects that a higher level of education would lead to an increase in the woman’s negotiating skills and power and that this would contribute to a lower risk of SVWM. However, this was not the case for our study population. This result was similar to the findings reported in our earlier paper in which women’s education was not associated with violence in general in the past 12 months [37]. On the other hand, the result was inconsistent with another study which showed that women’s educational status was significantly associated with women’s experience of violence [36]. Second, women with knowledge of sexual matters prior to marriage were at greater risk of SVWM. This is in contrast to the findings from a previous study [9]. We explored our data for other potential confounders such as women who knew about sex being more likely to report violence. However, we did not find any evidence for this. Therefore, this issue requires further investigation. Third, only one of the three community level variables (women’s autonomy) included in our analysis was significantly associated with SVWM. This highlights the importance of women’s autonomy, and that the advantages of increased gender parity in any individual household may spread to the surrounding community. Conversely, the lack of a community level education effect suggests that SVWM is mainly influenced by the education of individual men.

This study has some limitations. First, we cannot rule out the possibility of underreporting associated with reluctance to report highly sensitive experiences. However, data were collected by highly experienced interview teams using a carefully designed questionnaire and with extensive training and supervision. Thus, the observed findings are unlikely to be attributable to differential levels of reporting. Second, the cross-sectional nature of the data erodes our ability to establish temporality or causality in the observed relationships. It is possible that the observed relationships work in the reverse direction or that the outcomes are caused by unmeasured intermediate factors. In order to study the causes of sexual violence a panel study following women through the course of their marriage would be necessary.

These limitations notwithstanding, our analysis makes several new and important contributions to the literature on the determinants of SVWM in developing countries. First, it has identified a number of individual, households-level socioeconomic and interpersonal factors that are strongly predictive of the risk of SVWM. Second, it has reaffirmed the pivotal role of women’s autonomy both at an individual and a community level. Third, it has shown that women’s education actually plays a less significant role compared to other individual, household and interpersonal-level characteristics. Fourth, it has highlighted the importance of husband’s education as a protective factor for SVWM for young women.

## Conclusions

This study has shown that SVWM among young women in Nepal is common. The determinants of SVWM are complex. However, women’s autonomy, education of husbands, interpersonal communication and alcohol use of husbands were all highly related to SVWM. In particular, the study highlights the critical importance of women’s autonomy at both the individual and community level. Some of the critical interventions to enhance women’s autonomy and rights, improving their economic status, and addressing gender norms and practices would, therefore, benefit from strong community level efforts. As a priority, program managers and policy makers must develop strategies that increase women’s autonomy, involve men and educate them on gender issue and encourage them to engage in inter-spousal communication. It should also be remembered that even though women’s education did not have a statistically significant relationship with SVWM, education of women could be used as a tool to improve women’s autonomy; the fact that education was not found to be significantly associated with sexual violence indicates that education of women in Nepal may need to be refocused so as to provide women with the life skills they need.

## Authors’ contributions

MP conceived and designed the study. He monitored and supervised the data collection, analysed the data, and prepared the manuscript. MF assisted in the analysis of the data and preparing the first draft of the manuscript. JT developed study instruments, and was involved in training of researchers and monitored the data collection. PL reviewed and revised the paper for its content. IS reviewed the paper and contributed to revising it critically for intellectual content. All authors read and approved the final content of the manuscript.

## Competing interests

The authors declare that they have no competing interests.

## References

[B1] World Health Organisation World Report on Violence and Health 2002 World Health Organisation, Geneva

[B2] Zierler S Feingold L Laufer D Adult survivors of childhood sexual abuse and subsequent risk of HIV infection Am J Public Health 1991 81 5 572 575 10.2105/AJPH.81.5.572 2014856 PMC1405069

[B3] Garcia-Moreno C Watts C Violence against women: its importance for HIV/AIDS prevention AIDS 2000 14 Suppl3 253 265 11086869

[B4] Maman S Intersection of HIV and violence: direction for future research and interventions Soc Sci Med 2000 50 4 459 478 10.1016/S0277-9536(99)00270-1 10641800

[B5] Watts C Mayhew S Reproductive health Services and intimate partner violence; Shaping a pragmatic response in Sub-Saharan Africa Int Fam Plan Perspect 2004 30 40 207 213 15590387 10.1363/3020704

[B6] Ganju D Jejeebhoy S Nidavoluv V Santhya KG Finger N Forced Sexual Relation among Married Young Women in Developing Countries 2003 Paper presented at Non-consensual Sexual experiences of Young People in Developing Countries: A Consultative Meeting, New Delhi, India

[B7] World Health Organisation WHO Multi-Country Study on Women’s Health and Domestic Violence against Women 2005 World Health Organisation, Geneva

[B8] Puri M Cleland J Assessing the factors sexual harassment among young female migrant workers in Nepal J Interpers Violence 2007 22 11 1363 1381 10.1177/0886260507305524 17925287

[B9] Puri M Shah I Tamang J Exploring the nature and reasons associated with sexual violence within marriage among young women in Nepal J Interpers Violence 2010 25 10 1873 1892 10.1177/0886260509354514 20139347

[B10] Santhya KG Haberland N Ram F Sinha RK Mohnaty SK Consent or Coercion: Examining unwanted sex among married young women in India Int Fam Plan Perspect 2007 33 3 124 132 10.1363/3312407 17938095

[B11] Acharya R Koenig MA Sinha RK Prevalence and risk factors for sexual coercion against young married women by intimate partners: New evidence from rural India 2005 A paper presented at the XXVth IUSSP International Population Conference, Tours, France

[B12] Jejeebhoy S Bott S Jejeebhoy S, Shah I, Thapa S Non-consensual Sexual experiences of Young People in Developing Countries: An Overview Sex without consent: Young people in developing countries 2005 Zed Books, New York

[B13] Koenig M Zabolkota I Lutalo T Nalugoda F Wagmen J Gray R Coerced first intercourse and reproductive health among adolescent women in Rakai, Uganda Int Fam Plan Perspect 2004 20 4 156 163 15590381 10.1363/3015604

[B14] Im-Em W Kanchanachitra C Archavanitkul K Jejeebhoy S, Shah I, Thapa S Sexual coercion among ever-partnered women in Thailand Sex without consent: Young people in developing countries 2005 Zed Books, New York

[B15] Heise L Ellsberg M Ending Violence against Women. Population reports series L Issues World Health 1999 11 1 43 11056940

[B16] Santhya KG Jejeebhoy S Jejeebhoy S, Shah I, Thapa S Young women’s experiences of forced sex within marriage: evidence from India Sex without consent: Young people in developing countries 2005 Zed Books, New York

[B17] Population Council The Adverse Health and Social Outcomes of Sexual Coercion: Experiences of young women in developing countries 2004 YouthNet.Population Council/World Health Organisation,

[B18] Jejeebhoy S Santhya KG Forced Sex within Marriage among Young Women: Evidence from South Asia 2003 Paper presented in a consultative meeting on non-consensual sexual experiences of young people in developing Countries, New Delhi, India 22 25

[B19] Choe M Thapa S Mishra V Early marriage and early childbearing in Nepal J Biosoc Sci 2004 00 1 20 15768770 10.1017/s0021932003006527

[B20] Khan ME Townsend JW D’Costa S Behind closed doors: A qualitative study on sexual behaviour of married women in Bangladesh Cult Health Sex 2002 4 2 237 256 10.1080/13691050110102253

[B21] Outtara M Sen P Thompson M Forced marriage, Forced sex: The perils of childhood for girls Gend Dev 1998 6 3 27 33 10.1080/741922829 12294409

[B22] George A Embodying identity through heterosexual sexuality-newly married adolescent women in India Cult Health Sex 2002 4 2 207 222 10.1080/13691050110095856

[B23] Joshi AM Dhapolam E Experiences and perceptions of marital sexual relationships among rural women in Gujrat, India Asia-Pac Popul J 2001 16 2 177 194

[B24] Hoffman K Demo DH Edwards JN Physical wife abuse is a non-Western society: An integrated theoretical approach J Marriage Fam 1994 56 131 146 10.2307/352709

[B25] Nelson E Zimmerman C Household survey on domestic violence in Cambodia 1996 Ministry of Women’s Affairs and the Project against Domestic Violence, Phnom Penh, Cambodia

[B26] Parish WL Wang T Laumann EO Pan S Luo Y Intimate partner violence in China: National prevalence, risk factors and associated health problems Int Fam Plan Perspect 2004 30 4 174 181 10.1363/3017404 15590383

[B27] Rao V Wife beating in rural South India: A qualitative and econometric analysis Soc Sci Med 1997 44 8 1169 1180 10.1016/S0277-9536(96)00252-3 9131741

[B28] Koenig M Ahemed S Hossain M Mozumder ABM Women’s status and domestic violence in Rural Bangladesh: Individual-and-community level effect Demography 2003 40 2 269 288 10.1353/dem.2003.0014 12846132

[B29] Pradhananga RB Shrestha P Domestic Violence against Women in Nepal: Concept, History and Existing Laws 2010 Forum for Women Law and Development, Kathmandu, Nepal [http://www.childtrafficking.com/Docs/domestic_violence_0607.pdf], Accessed 28 June 2010

[B30] Puri M Tamang J Shah I Dulal B Donta B, Shah I, Puri CP Investigating Nature and Causes of Sexual Violence during Early Years of Marriage in Nepal Gender-based Violence and Sexual and Reproductive Health 2010 National Institute for Research in Reproductive Health and World Health Organization and Indian Society for the Study of Reproduction and Fertility, Mumbai, India 207 224

[B31] Pokharel S Kulozyoki A Shakya S School-Based Sex Education in Western Nepal: Uncomfortable for both Teachers and Students Reprod Health Matters 2006 14 28 156 161 10.1016/S0968-8080(06)28255-7 17101434

[B32] Bennett L Dangerous Wives and Sacred Sisters. Social and symbolic Roles of High caste women in Nepal 1983 Columbia University Press, New York

[B33] Cameron M On the edge of the auspicious: Gender and Caste in Nepal 2005 University of Illinois Press, Chicago and Mandala Publications, Kathmandu

[B34] Government of Nepal The Domestic Violence and Punishment Act 2065 2009 Government of Nepal, Kathmandu, Nepal

[B35] Puri M Hawkes S Tamang J Bhattarai A Gender-based Violence among women in selected districts of Nepal 2012 Center for Research on Environment Health and Population Activities and The Asia Foundation, Kathmandu, Nepal

[B36] Adhikari R Tamang J Sexual coercion of married women in Nepal BMC Womens Health 2010 10 31 10.1186/1472-6874-10-31 21029449 PMC2987890

[B37] Lamichhane P Puri M Tamang J Dulal B Women’s Status and Violence against Young Married Women in Rural Nepal BMC Womens Health 2011 11 19 10.1186/1472-6874-11-19 21612603 PMC3121674

[B38] Clogg CC Some Models for the Analysis of Association in Multiway Cross-Classifications Having Ordered Categories J Am Stat Assoc 1987 77 803 815

[B39] McCutcheon AL Latent Class Analysis Sage University Paper Series on Quantitative Applications in the Social Sciences 1987 Sage, Newbury Park, CA

[B40] Jejeebhoy S Convergence and Divergence in Spouses’ Perspectives on Women’s Autonomy in Rural India Stud Fam Plann 2002 33 4 299 308 10.1111/j.1728-4465.2002.00299.x 12553187

[B41] Collins LM Wugalter SE Fidler PL Eye AV, Clogg CC Some Practical Issues Related to the Estimation of Latent Class and Latent Transition Parameters Categorical Variables in Development Research: Methods of Analysis 1996 Academic, San Diego

[B42] Manley BFJ Multivariate statistical methods 1994 Chapman and Hall, London

[B43] Gwatkin DR Rutstein S Johnson K Suliman E Wagstaff A Socio-economic differences in health nutrition and population in Nepal 2000 World Bank, Washington 1 27 18293634

